# Supplementary material for: Cloud BioLinux: pre-configured and on-demand bioinformatics computing for the genomics community
Source: BMC Bioinformatics. 2012 Mar 19;13:42. doi: 10.1186/1471-2105-13-42 (PMC3372431; doi:10.1186/1471-2105-13-42)
Supplement: Additional file 1 — Supplementary 1 Cloud BioLinux software documentation in the form of a mini, self-contained website. Users need to download and uncompress the .zip file, and open through a web browser the "index.html" file available on the main directory. (ZIP 1823 kb). [file 1471-2105-13-42-S1.ZIP › Cloud-BioLinux-Package-Documentation/docs/Taverna.html]

Bio-Linux Software Documentation Pages

Back to search form

## Taverna

|  |  |
| --- | --- |
| Name | Taverna |
| Description | **Taverna** is a graphical client for setting up workflows that make use of web services. Taverna allows the user to graphically develop workflows, and the default configuration provides a simple view and access to many bioinformatics web services currently available from various sites around the world. In other words, Taverna will allow those with limited computing background and limited technical resources to construct complex analyses using public and private data. Workflows can be saved for re-use or to share with others.  **Taverna** is a component of the myGrid project. Further information about Taverna is available at http://taverna.sourceforge.net The myGrid project homepage is at: http://www.mygrid.org.uk |
| Homepage | http://taverna.sourceforge.net |
| Remote Documentation | http://taverna.sourceforge.net/index.php?doc=docroot.html |

Bio-Linux specific notes
